# Approaches to optimising access to NICE-approved biologic anti-TNFs for patients with rheumatoid arthritis with moderately active disease

**DOI:** 10.1186/s12916-023-02746-5

**Published:** 2023-02-14

**Authors:** Peter C. Taylor, Ayman Askari, Ernest Choy, Michael R. Ehrenstein, Sara Else, Muhammad K. Nisar

**Affiliations:** 1 Botnar Research Centre, Nuffield Department of Orthopaedics, Rheumatology and Musculoskeletal Sciences, University of Oxford, Windmill Road, Headington, Oxford, OX3 7LD UK; 2 Robert Jones and Agnes Hunt Orthopaedic and District Hospital NHS Trust, Oswestry, UK; 3 Department of Infection and Immunity, Cardiff University, Cardiff, UK; 4 Centre for Rheumatology, UCL Division of Medicine, Rayne Building, 5 University Street, London, UK; 5 NHS Forth Valley, Stirling, UK; 6 Luton & Dunstable University Hospital, Lewsey Road, Luton, UK

**Keywords:** Moderate rheumatoid arthritis, Biologics, Biosimilars, National Institute for Health and Care Excellence (NICE), Telemedicine

## Abstract

**Background:**

Rheumatoid arthritis (RA) is a chronic inflammatory disease that is associated with joint pain and stiffness. Biologics represent some of the most effective treatments for RA, but previous guidance from the National Institute for Health and Care Excellence (NICE) has limited their use to patients with severely active disease. This has meant patients with moderately active RA have been treated as if they have an acceptable disease state, despite many cases where the inflammation has a major impact on joint damage, mobility, pain and quality of life. However, recent guideline changes (NICE TA715) have approved the use of three biologics — adalimumab, etanercept and infliximab — for the treatment of moderately active RA.

**Main body:**

In response to these changes, we have held discussions with medical teams from across the UK to consider the main implications for implementation of these new recommendations, as well as any differences in approach that may exist at a local level. Several key challenges were identified. These included establishing methods of educating both physicians and patients concerning the new availability of the biologic treatments, with suggestions of various organisations that could be approached to circulate informative material. Identifying which patients with moderately active RA stand to benefit was another discussion topic. Relying solely on scoring systems like Disease Activity Score in 28 Joints (DAS28) was acknowledged to have limitations, and alternative complementary approaches such as ultrasound, as well as assessing a patient’s co-morbidities, could also be useful tools in determining those who could benefit from biologics. An additional challenge for the process of patient identification has been the increase in the use of telemedicine consultations in response to the coronavirus disease 2019 (COVID-19) pandemic. More use of patient-reported outcomes was raised as one possible solution, and the importance of maintaining up-to-date databases on patient disease scores and treatment history was also stressed.

**Conclusion:**

While challenges exist in education and identifying patients who may benefit from the use of biologics, the NICE TA715 recommendations hold great potential in addressing an unmet need for the treatment of moderate RA.

## Background

Rheumatoid arthritis (RA) is a chronic inflammatory disease that is estimated to affect ~400,000 people in the United Kingdom (UK) [1]. The disease is characterised by joint swelling and stiffness caused by underlying inflammation, with additional symptoms including pain, fatigue and depression. Given that the most rapid period of joint damage occurs within the first 2 years of RA onset [2], timely treatment interventions are crucial to minimise the impact of the disease. In addition to joint damage, failure to effectively treat RA leads to profound difficulties in numerous other aspects of a patient’s quality of life [3].

International recommendations for the treatment of RA emphasise early intervention with conventional synthetic disease-modifying anti-rheumatic drugs (csDMARDs), with an ideal treatment target of remission or, where that is not achievable, low disease activity. Achieving rapid remission or low disease activity is the best means to prevent joint damage and improve the patient’s quality of life [4–8]. If the therapeutic target is not attained within 6 months of treatment, then advanced therapies such as biologic anti-tumour necrosis factors (TNFs) are recommended.

Over the last two decades, biologic TNF inhibitors have transformed achievable outcomes for patients with a wide variety of immune-mediated inflammatory diseases, including RA. Biologics are proteins that can block a key component of the inflammation response, such as TNF, that normally triggers joint swelling and other symptoms. However, the very high acquisition costs of biologic originators resulted in National Institute for Health and Care Excellence (NICE) guidance that restricted their access to only those patients with high disease activity [9, 10]. When originator drugs approached patent expiry, biosimilar drugs emerged, and several have been approved for use in Europe. The first to be approved were infliximab and etanercept biosimilars and more recently adalimumab biosimilars. The European Medicines Agency requires that biosimilars are highly similar to the original biologic and display no clinically meaningful differences in terms of safety, quality and efficacy [11].

The introduction of the less expensive biosimilars holds promise for reducing the treatment costs of the biologics, while also providing a similar efficacy on RA disease management. Indeed, in a study in the Department of Rheumatology in Bernhoven, it was reported that the introduction of an etanercept biosimilar led to a significant decrease in the average rheumatic medication cost per patient in its first quarter (−€370), followed by a decrease in the quarterly trend of average rheumatic medication cost after this (−€50.34). However, this economic advantage was offset by an increase in the number of overall patients being treated with biologics [12]. A similar conclusion was made from a UK study [13].

### Changes to NICE guidelines

In the UK, the NICE has guided the National Health Service (NHS) in England and Wales for the optimal pathway used to treat patients. The guidelines themselves are designed to ensure the cost-effectiveness of drugs purchased by the NHS, based on considerations concerning the efficacy according to both disease severity and procurement price. Consequently, for over two decades, this has led to a system in which some of the most effective treatments for people living with RA (i.e., biologics) have been restricted to patients with severely active diseases that have failed to respond adequately to two csDMARDs, one of which must have included taking methotrexate for at least 6 months. Similar restrictions have been in place in Scotland. Limiting biologic access to solely severe RA has, in effect, meant that for a generation, patients living with moderately active RA have been treated as if this was an acceptable state. The reality is often far from this. The impact on mobility, pain and quality of life on patients with moderate RA can be major, and there is a clear unmet need for these individuals [1]. Notably, patients with moderate RA have been shown to respond well to biologics, and they are in fact more likely to achieve and sustain remission states, with all the accompanying advantages of improved employment prospects, and reduced long-term requirement for orthopaedic intervention [5, 14].

The costs associated with biosimilars have declined significantly in recent years. Owing to the reduction in prescription costs, recent NICE guidance (TA715) has been updated to allow the use of three biologic anti-TNFs — namely adalimumab, etanercept and infliximab — for use in patients with moderately active RA who have not responded to two or more conventional csDMARD therapies, if the companies provide them at the same or lower prices than those agreed with the Commercial Medicines Unit. Abatacept was also included in TA715 but is not recommended, within its marketing authorisation, for treating moderate active RA in adults when one or more csDMARDs have not controlled the disease well enough [15]. Of note, adalimumab and etanercept can be used in monotherapy in patients who cannot take methotrexate because of toxicity or tolerability issues. With the relaxation of NICE guidelines, it is estimated that up to 25,000 patients with moderate RA will now be able to benefit from, and be eligible to receive, these biologic treatments [16]. However, the process for identifying these candidates remains to be elucidated.

Unlike the UK, the use of biologics and biosimilars for the treatment of RA with moderate disease activity has previously been approved in other territories, including Europe and the USA. For Europe, guidelines from the European League Against Rheumatism (EULAR) advise anti-TNFs to be started if treatment with methotrexate and glucocorticoids leads to no improvement in a patient’s RA following 3 months and the target of remission or low disease activity is not achieved within 6 months and if the patient presents with poor prognostic factors. Further guidance on drug switching and cycling for when an anti-TNF has failed is also provided. The guidelines suggest that drugs that are less costly should be preferred over more costly ones as long as they are similarly efficacious and safe [7]. The introduction of biosimilars has resulted in an increase in the number of patients receiving biologic therapy for RA in some countries [12]. In the USA, the American College of Rheumatology include conditional recommendations on the use of anti-TNFs [8]. For example, they advise methotrexate as a monotherapy in treatment-naive patients, owing to the higher cost of anti-TNFs and their risk of toxicity. However, the guidelines acknowledge anti-TNFs are associated with a more rapid onset of action and a greater chance of improvement, so their use may be preferable for some patients with poor prognostic features. Therefore, these considerations may be important to take into account in the future development of biologic and biosimilar guidelines in the UK. Although the focus of this manuscript is on the UK, the challenges associated with identifying patients who may benefit from biologic therapy remain a universal issue.

### Telemedicine and care following COVID-19

The coronavirus disease 2019 (COVID-19) pandemic continues to challenge our healthcare system and has impacted how we care for patients in the UK; moreover, it has also emphasised differences in care across post codes [17]. When government restrictions on movement and activity were in place to reduce COVID-19 spread, it affected all aspects of the daily life of the general population. Those within more rural communities have become more isolated, and patients became less likely to seek in-person care in larger cities, even if they experienced a change in their RA symptoms. For instance, instead of travelling more than 2 h using public transport on top of the time taken to wait for their appointment, many patients opted for virtual medical appointments and continue to express that preference despite the relaxation of restrictions. In other circumstances, owing to changes that affect working routines or employment status, patients may no longer have the opportunity to take time off to be seen in the clinic and so prefer a virtual medical appointment. It should be noted that in more recent months, the number of face-to-face appointments is increasing again, although perhaps not to the same extent as they were pre-pandemic. However, there are some patients for whom remote consultations are unsuitable. Furthermore, factors, such as language barriers, older age, limited access or ability to use technology, disadvantaged socio-economic or educational status, or difficulties of hearing, cognition, or speech, can all limit the use of telemedicine [18]. For these patients, face-to-face appointments are necessary.

At the beginning of the pandemic, one option open to rheumatologists conducting remote consultations was to use short-term steroids as a means to treat symptomatic patients until they could be seen at a face-to-face review, while being mindful of the potential risks associated with corticosteroid use [19]. At the time of writing, rheumatology services around the nation are dealing with a prolonged backlog of clinical care, and telemedicine reviews continue to be employed for selected patients. This change in practice has potential implications for the identification of people with moderately active RA who might now be eligible for treatment with biologic therapies in line with NICE TA715 recommendations, and also for timely intervention.

### Recommendations and methodology

Following the release of the NICE TA715 recommendations [15], we have held five focus group discussions with medical teams from across the UK (London, Scotland, Wales, West Midlands and East of England), to review the main challenges and implications surrounding these updates. Groups comprised consultant rheumatologists, clinical nurse specialists, rheumatology specialist nurses, and rheumatology, biologics and specialist pharmacists. The authors are aware that similar discussions have taken place separately within patient charities, but the present report is restricted to the findings and recommendations of the national focus groups. Based on some of the most common discussion topics in these meetings, we developed four questions:

1. What recommendations can be suggested to help educate patients, physicians, nurses and other healthcare professionals of the new opportunities for RA treatment based on changes to NICE guidelines?
2. How will the cohort of eligible patients with moderately active RA be identified, and what particular characteristics will suggest that targeted therapies will or will not be beneficial?
3. In cases of patients with moderately active RA who are accepting of their condition, what approaches can be taken to encourage them to consider the novel treatment opportunities?
4. How will patients with moderately active RA who predominately have care through remote appointments be identified for the novel treatments?

**Fig. 1:**
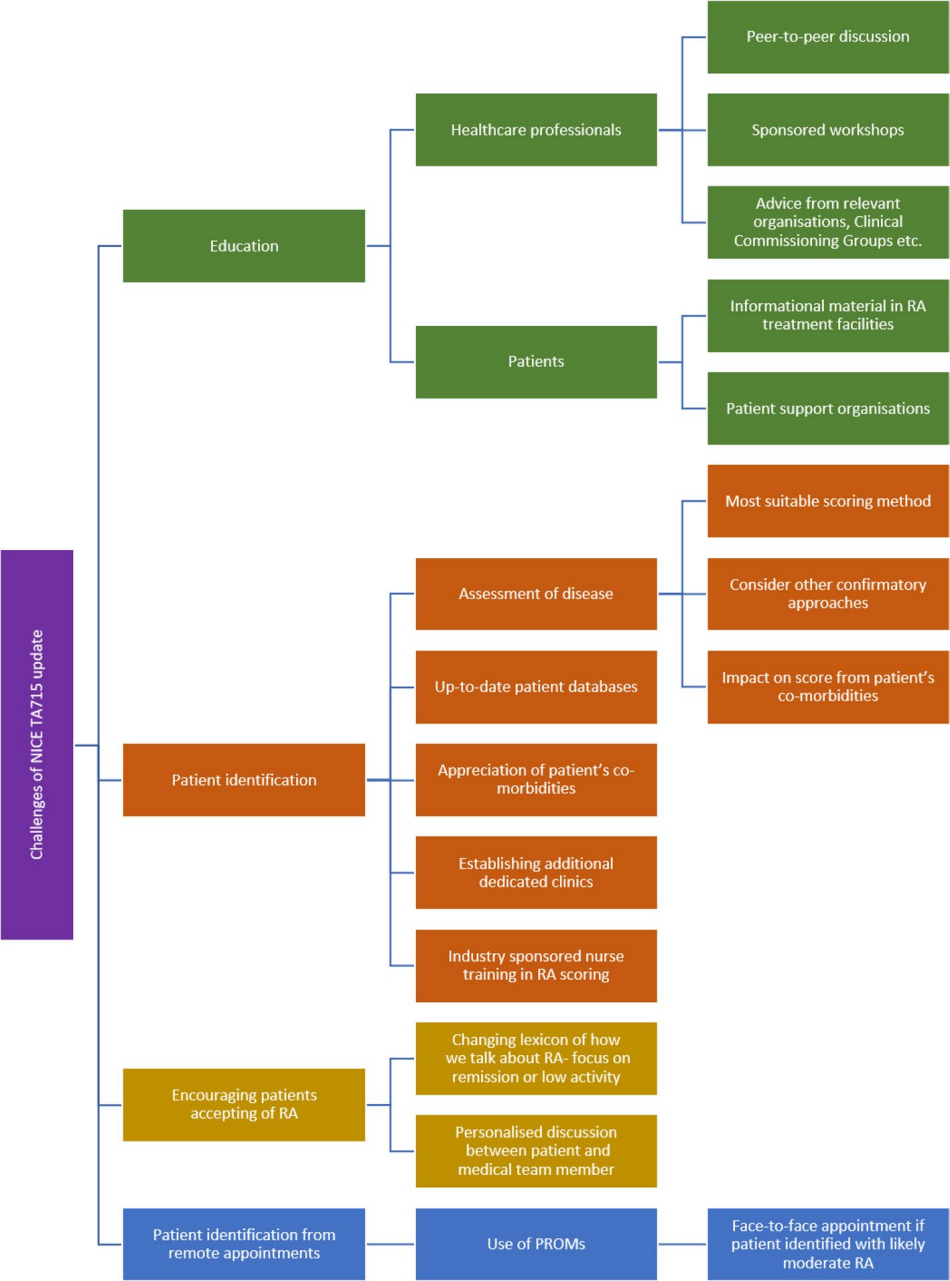
Summary of the main challenges surrounding the implementation of the NICE TA715 guideline updates. Abbreviations: NICE, National Institute for Health and Care Excellence; PROM, patient-reported outcome measure; RA, rheumatoid arthritis

These questions were circulated to all authors, and the discussion that followed was used as the basis for the rest of this manuscript. The main responses are summarised in Fig. 1.

### Education of guideline changes

Ensuring healthcare professionals and patients are made aware of the opportunities afforded by the NICE TA715 recommendations will be an important step in their implementation. Appropriate education for medical staff and patients will require different approaches and is anticipated to benefit from the co-operation of societies, organisations and industry.

Awareness of the TA715 recommendations amongst rheumatologists will likely be best facilitated through peer-to-peer discussion at national rheumatological meetings such as The Scottish Society of Rheumatology and The British Society for Rheumatology. Additionally, NICE themselves could play a key role in educating healthcare professionals via circulars, seminars and sponsored workshops, outlining the major changes of the guideline update.

Organisations and commissioning groups can play a similar role in providing advice and guidance to medical teams. This is already underway in some regions of the UK. In Scotland, Healthcare Improvement Scotland have distributed advice via Medical Directors to all Health Boards indicating that the recommendations are as valid for Scotland as for England and Wales. This in turn has been passed to Clinical Leads and teams for discussion and implementation. Elsewhere, Clinical Commissioning Groups have engaged with hospital trusts to inform them of guideline updates. Likewise, Regional Medicines Optimisation Committees could provide educational support to pharmacists where needed, highlighting the availability of adalimumab, etanercept and infliximab for patients with moderately active RA.

Effective methods to inform patients of the new treatments available through the NICE TA715 recommendations are perhaps less clear. Some patients with moderately active RA and troublesome symptoms are anticipated to come forward rapidly on hearing news of the new guidance regarding the change in eligibility for access to a targeted therapy. But others, predominantly those who may have lived with the consequences of moderately active disease for many years, may be accepting of their symptomatology and will perhaps also have concerns about any potential risks associated with a more potent therapeutic regime.

Producing educational material highlighting the key messages of the NICE TA715 recommendations will be important in spreading awareness to patients and could be developed and circulated through collaboration between health care professionals, patient support organisations and NICE themselves. Such material should focus on aspects such as the main guideline changes and the evidence backing them, support of the efficacy of biologics in RA, and which patients are now eligible, using appropriate language. As with all pharmacological interventions, a careful explanation of the likelihood and nature of benefits and any potential risks of biologic treatment is essential in order to facilitate fully informed, shared decision-making regarding treatment choice. Displaying this print media in RA treatment facilities, such as treatment waiting areas, will target those patients who stand to benefit from the update. This can help both general practitioners and patients to consider the impact of their RA, and urge them to discuss their therapy with their specialists. Particular attention will need to be paid to address those patients where language and communication remain a barrier, as well as those living in poverty. There is an opportunity here for proactive outreach to ethnic communities. For example, the National Rheumatoid Arthritis Society engage with the South Asian community through their ‘Apni Jung’ (translates to ‘Our Fight’ in Hindi) initiative.

Patient support organisations, such as the National Rheumatoid Arthritis Society or Arthritis and Musculoskeletal Alliance, represent a strong platform for spreading awareness online. These groups are well trusted by patients and can play a pivotal role in advertising widely and sharing case stories of successfully treated patients, as well as hosting events such as interactive seminars. The use of other mediums, including podcasts and ethnography projects, can help enhance confidence amongst people with RA, particularly those who may have accepted their moderate disease and would have lost the will, hope or even trust in the treatment or their treating physicians. In addition to producing their own material, patient support groups can also signpost patients to relevant sources and educational tools. This activity will help to reassure patients and also save time for health care professionals by resolving queries patients may have.

### Identification of candidate patients

Identifying those patients with moderate RA who stand to benefit from the new availability of the biologics now approved under TA715 represents a major challenge, which is compounded further by restrictions and backlogs brought by the COVID-19 pandemic. Ideally, establishing dedicated clinics for the assessment of disease activity will enable the greatest number of patients to be evaluated, but this may not be feasible given the already overstretched status of NHS rheumatology services. There may be opportunities to seek industry sponsorship for funding of nurses trained in the assessment of disease activity assessments in order to help facilitate the necessary additional clinics. In such cases, however, to ensure parity of access to all available treatment options, it will be important to establish an appropriate alignment of the aims of all involved parties. The ideal and recommended treatment target of remission, with correspondingly optimum quality of life, is generally more readily achieved when biologic anti-TNFs are initiated while a patient is in moderate disease activity rather than in high disease activity, and when they are earlier in their disease course. Contemporary treatment recommendations [7] recognise that for a high proportion of those patients achieving a sustained remission on biologic anti-TNF, for 6 months or longer, it will be possible to taper the biologic dose while maintaining remission status on csDMARDs. This approach is designed not only to optimise benefit to risk, but also to ensure the most cost-effective use of resources for the wider health economy. Therefore, it is key to develop robust and effective methods for the identification of people living with RA with moderately active disease despite treatment for 6 months or more with csDMARDs. The creation and maintenance of up-to-date patient databases seem the most powerful tool through which to monitor and track patients. Storing information regarding overall patient numbers, as well as individual disease activity scores and their current and prior treatments, will be the best guide to select candidates for the new biologics. At present, there seems to be a degree of disparity across the UK regarding access to suitable databases and the resources required to develop, maintain and populate them. This raises the issue of a post code lottery existing, with those patients in regions without reliable databases not being identified as quickly, and at risk of delays in optimising treatment. Therefore, there is clearly a need for support at a national level to implement improved databases and software where needed.

Of course, the question remains as to how best to measure and record a patient’s RA disease activity state. Many physicians still rely heavily on the Disease Activity Score in 28 Joints (DAS28), with moderate RA characterised as a score >3.2 and ≤ 5.1. While widely used, this method can have issues with consistency and has its own limitations. For example, there are differences between the use of DAS28 using erythrocyte sedimentation rate (DAS28-ESR) and C-reactive protein (DAS28-CRP) in terms of interpretation of the measure. A DAS28-CRP underestimates disease activity in RA by comparison with DAS28-ESR [20].

A careful clinical assessment will be necessary to ensure that the likelihood of benefit is greater than the risk of toxicity on an individual patient basis, and in shared decision-making that this is acceptable to the patient. For example, not all patients with an elevated disease activity score in the moderate or even high range will necessarily benefit from biologic treatment. Interpretation of an elevated score may be confounded by co-morbidities such as fibromyalgia, osteoarthritis and insomnia. In such cases, there are concerns that a composite score may overestimate inflammatory disease activity in patients with chronic pain. Indeed, this increases the risk of individuals who are unlikely to benefit from treatment escalation and being exposed to unnecessary harm as a consequence of exposure to potentially immunosuppressive medication. In contrast, current non-biologic therapies may lower the score while having a high potential for detrimental toxicity, such as osteoporosis, when used in the long term [21]. This is especially true of concomitant steroid treatment, which has sometimes been used in symptomatic patients with moderately active disease in UK practice as a consequence of past NICE guidance which restricted eligibility for advanced therapies [22]. The latest update of EULAR recommendations for the management of RA, based on a comprehensive systematic literature review of evidence [23], emphasises the importance of tapering and discontinuing steroids because of the risks of toxicity, although their short-term use as a bridging therapy when initiating csDMARDs remains a recommendation as in previous iterations of the management recommendations. Nonetheless, the recently published findings of a pragmatic double-blind, randomised trial, comparing 2 years of prednisolone (5 mg/day) to placebo in patients aged 65 or more with active RA, concluded that the beneficial effects in terms of disease activity improvement justified the 24% increase in adverse events, most of which were non-serious [24].

An advantage of optimising disease control with a biologic anti-TNF is that steroids can be tapered and gradually withdrawn. In cases of doubt regarding the contribution of synovitis to the disease activity score assessment, as may occur for example in obese patients, the use of other confirmatory methods, including ultrasound imaging, to establish the presence of inflammatory disease could help provide a more reliable means of identifying those patients most likely to benefit.

The presence of co-morbidities may also be an important consideration in determining the patients who stand to benefit most from the new eligibility for biologic therapies recommended in NICE TA715. Since anti-TNFs such as adalimumab, etanercept and infliximab are known to reduce cardiovascular co-morbidity [25–28], they may represent a treatment of choice in patients with moderately active RA with early or established cardiovascular disease, but without advanced stage heart failure [29]. In the case of overweight patients, a high body mass index can be accompanied by an increase in acute phase markers and in DAS28 score; therefore, referral to a 12-week gym programme and signposting healthy eating websites may be a more desirable first step as opposed to moving straight to biologics. Indeed, a higher body mass index has been previously suggested as a predictor of poorer response to anti-TNF [30–32] and higher pain following TNF-α inhibitor treatment [33], thereby emphasising the importance of education regarding weight optimisation [34].

Other lifestyle choices, and in particular smoking, may also have a detrimental influence on otherwise achievable outcomes with biologic treatments. For example, smoking has been associated with a reduced response to infliximab treatment [35, 36]. Therefore, with regard to health benefit in general, and optimising response to biologic anti-TNFs in particular, it is important to counsel people living with RA to stop smoking or at least to reduce smoking behaviour.

After identifying a candidate patient, it will also be necessary to consider the logistical issues and convenience to the individual with respect to subcutaneous versus intravenous treatment administration, given that there is an intravenous formulation of infliximab.

Although the present manuscript specifically concerns TA715, it should be noted that for those patients unable or unwilling to receive parenterally administered advanced therapies, NICE have also approved two orally available small-molecule Janus kinase inhibitors for use in RA patients with moderately active disease: filgotinib (TA676, [37]) and upadacitinib (TA744, [38]). Being ‘small molecules’, Janus kinase inhibitors are not immunogenic, unlike biologic anti-TNFs where immunogenicity is likely to account for much of the observed loss of treatment response over time [39]. This is particularly problematic in patients on biologic anti-TNFs who are intolerant of concomitant methotrexate or who are not adherent to their prescribed methotrexate regime.

### Addressing patients who are accepting of their rheumatoid arthritis condition

Addressing those patients who are more accepting of their RA condition will not be an easy matter to resolve. The best approach here will likely involve direct discussion between the patients and medical team members with a personalised assessment of the likelihood of benefit and possibility of risk and means to mitigate any such risks. This could take the form of establishing advanced or practice nurse-delivered clinics. Such clinics would serve to highlight the risks associated with ongoing disease, with the goal of agreeing on the best treatment course through a shared decision-making process. In the case of both patients and physicians who have become habituated to the concept of ‘stable’ but moderately active disease, perhaps even changing the lexicon may encourage consideration of a change in disease management. For example, the word ‘stable’ could be removed when referring to the management of moderately active disease, and clinical dialogue should be focussed instead on the goal of remission or low disease activity.

### Impact of COVID-19 on candidate identification

The COVID-19 pandemic has impacted medicine greatly, causing a large patient backlog, stretching of hospital resources and personnel and increasing the number of remote telemedicine clinics compared to face-to-face visits. Indeed, the restrictions imposed by the pandemic have limited the opportunity to review all patients with moderate RA disease activity as to their suitability to receive the biologics outlined in NICE TA715. It is imperative to provide fresh solutions to these challenges and establish robust practices to identify patients who may benefit from biologic therapy. The work of patient organisations has sought to highlight that any individual with moderately active disease may now benefit from the newly available biologic therapies, and they have encouraged patients to contact their rheumatology department for a review. Patient empowerment and engagement to take an active role on a treatment plan is essential [40]. This is facilitated by the use of patient-reported outcome measures (PROMs) for assessment of the aspects of life most impacted by RA. The use of PROMs also provides a supplementary means of scoring and monitoring RA disease activity and progression, whether a consultation is in person or by telemedicine. With a larger number of remote appointments and clinics, self-reported swollen and tender joint counts in suitably trained patients could be employed for DAS28 scoring [41]. However, there is some scepticism of the reliability of these measures when recorded by patients remotely, and the timeframe required for training patients to assess this properly could prove extensive. Other well-validated PROMs such as Rheumatoid Arthritis Impact of Disease (RAID) or Routine Assessment of Patient Index Data 3 (RAPID-3) can also be used for telemedicine [42] and generally track well with DAS28 [43]. Questionnaire-based PROMs, including the Standardised Health Questionnaire (EQ-5D) and Health Assessment Questionnaire (HAQ), may be viable options although these do not always align with DAS28 scoring. Further composite PROM systems, such as Patient-based Disease Activity Score (PDAS), have been shown to have the potential in predicting disease flare and treatment escalation [44]. Adapting PROMs into a mobile application could provide a quicker, more patient-friendly method of collecting data and keeping it regularly updated. Asking patients to submit PROM scores every 2 to 3 months would enable up-to-date tracking of a patient’s disease and help flag up when attending an in-person visit would be of use. Indeed, there is already evidence in support for mobile and web-based applications for patient self-assessment in RA [45–47].

Once a patient is identified with having likely moderate (or severe) RA through PROMs monitoring, a face-to-face appointment should be arranged as soon as possible. From here, formal disease activity assessment can be performed, and shared decision-making discussions regarding the likely benefits and any risks of biologic therapy can be initiated as appropriate.

## Conclusions

The recommendations under NICE TA715 provide great promise for improving outcomes, by widening access to biologic anti-TNF treatment to include patients with moderately active RA. The greatest challenges will be spreading awareness of the new treatment options afforded by this update to both medical staff and patients alike, as well as identifying those individuals who stand to benefit most from the new therapies. Support to provide and maintain effective patient databases will likely be the most effective means of identifying suitable patients, backed up by data collected from a combination of in-clinic disease scoring, joint imaging for inflammation and PROMs. Factors such as the extent of active inflammation and the presence of co-morbidities will also be important in determining those patients who are most likely to benefit.

The work and views described in this opinion piece were prompted by new guidance for the management of RA issued by NICE, which allows people living with moderately active RA access to effective biologic agents for the first time. While it is the case that the specific considerations with respect to NICE are primarily applicable to the UK, and as such represent a limitation of this work, NICE guidelines have been adapted and incorporated by other countries [48]. Furthermore, ensuring that eligible patients who will benefit from effective biologic intervention can actually access such treatment should be a priority for all healthcare providers, irrespective of geography. Thus, many of the considerations we have outlined are likely to be more widely applicable. Inviting representatives from countries with prior access to biologic therapies for moderate RA to share their first-hand experiences could be used in future discussion to fortify recommendations. The focus groups we convened, whose discussions were used as the basis of this manuscript, comprised health care professionals who were invited with a view to developing strategies that might be adopted in clinical care pathways. However, the important role played by patient charities was acknowledged and highlighted. Therefore, the absence of patient representation at the originally convened focus groups can be considered a limitation that should be addressed in future dialogue.

## Data Availability

Not applicable.

## Abbreviations

COVID-19: Coronavirus disease 2019
csDMARD: Conventional synthetic disease-modifying anti-rheumatic drug
DAS28: Disease Activity Score in 28 Joints
DAS28-CRP: Disease Activity Score in 28 Joints using C-reactive protein
DAS28-ESR: Disease Activity Score in 28 Joints using erythrocyte sedimentation rate
EQ-5D: Standardised Health Questionnaire
EULAR: European League Against Rheumatism
HAQ: Health Assessment Questionnaire
NHS: National Health Service
NICE: National Institute for Health and Care Excellence
PDAS: Patient-based Disease Activity Score
PROM: Patient-reported outcome measure
RA: Rheumatoid arthritis
RAID: Rheumatoid Arthritis Impact of Disease
RAPID-3: Routine Assessment of Patient Index Data 3
TNF: Tumour necrosis factor
UK: United Kingdom
